# Methyl 4-(benz­yloxy)-3-meth­oxy­benzo­ate

**DOI:** 10.1107/S1600536813025415

**Published:** 2013-09-18

**Authors:** Kai Wang, ChaoFan Ju, Jian Xiao, Qiang Chen

**Affiliations:** a High Technology Research Institute of Nanjing University, Changzhou 213162, Jiangsu, People’s Republic of China; b School of Petrochemical Engineering, Changzhou University & High Technology Research Institute of Nanjing University, Changzhou 213164, Jiangsu, People’s Republic of China

## Abstract

In the title compound, C_16_H_16_O_4_, the aromatic rings are almost normal to one another, making a dihedral angle of 85.81 (10)°. In the crystal, mol­ecules are linked by C—H⋯O hydrogen bonds, forming chains propagating along the *b*-axis direction. There are also C—H⋯π inter­actions present which link the chains, forming two-dimensional networks lying parallel to (102).

## Related literature

For details of the anti­cancer properties of the drug Cediranib {systematic name: 4-[(4-fluoro-2-methyl-1*H*-indol-5-yl)­oxy]-6-meth­oxy-7-[3-(pyrrolidin-1-yl)prop­oxy]quinazol­ine}, for which the title compound is an important inter­mediate in the synthesis, see: Folkman (1996 ▶). For the synthetic procedure, see: Li & Zhang (2012 ▶). For bond-length data, see: Allen *et al.* (1987 ▶).
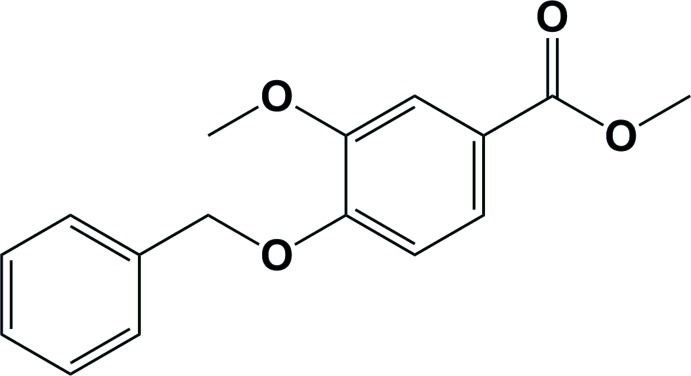


## Experimental

### 

#### Crystal data

C_16_H_16_O_4_
*M* *_r_* = 272.29
Monoclinic, 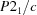

*a* = 5.2466 (7) Å
*b* = 17.973 (2) Å
*c* = 14.8785 (18) Å
β = 94.018 (3)°
*V* = 1399.6 (3) Å^3^
*Z* = 4
Mo *K*α radiation
μ = 0.09 mm^−1^
*T* = 293 K
0.12 × 0.12 × 0.10 mm

#### Data collection

Enraf–Nonius CAD-4 diffractometer
Absorption correction: ψ scan (North *et al.*, 1968 ▶) *T*_min_ = 0.989, *T*_max_ = 0.991
7732 measured reflections
2454 independent reflections
1666 reflections with *I* > 2σ(*I*)
*R*_int_ = 0.029
3 standard reflections every 120 min intensity decay: 1%

#### Refinement

*R*[*F* ^2^ > 2σ(*F* ^2^)] = 0.045
*wR*(*F* ^2^) = 0.138
*S* = 0.91
2454 reflections
182 parameters
H-atom parameters constrained
Δρ_max_ = 0.18 e Å^−3^
Δρ_min_ = −0.17 e Å^−3^

### 

Data collection: *CAD-4 Software* (Enraf–Nonius, 1985) ▶; cell refinement: *CAD-4 Software*; data reduction: *XCAD4* (Harms & Wocadlo, 1995 ▶); program(s) used to solve structure: *SHELXS97* (Sheldrick, 2008 ▶); program(s) used to refine structure: *SHELXL97* (Sheldrick, 2008 ▶); molecular graphics: *SHELXTL* (Sheldrick, 2008 ▶); software used to prepare material for publication: *SHELXTL*.

## Supplementary Material

Crystal structure: contains datablock(s) I, wang. DOI: 10.1107/S1600536813025415/su2645sup1.cif

Structure factors: contains datablock(s) I. DOI: 10.1107/S1600536813025415/su2645Isup2.hkl

Supplementary material file. DOI: 10.1107/S1600536813025415/su2645Isup3.cml

Additional supplementary materials:  crystallographic information; 3D view; checkCIF report

## Figures and Tables

**Table 1:** Hydrogen-bond geometry (Å, °) *Cg* is the centroid of the C1–C6 ring

*D*—H⋯*A*	*D*—H	H⋯*A*	*D*⋯*A*	*D*—H⋯*A*
C14—H14*B*⋯O3^i^	0.96	2.53	3.379 (3)	147
C14—H14*A*⋯*Cg* ^ii^	0.96	2.75	3.519 (2)	137

Symmetry codes: (i) 
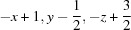
; (ii) 
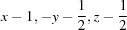
.

**Fig. 1.**
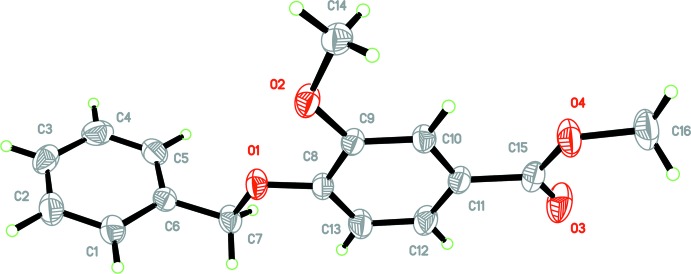
The molecular structure of the title molecule, with atom labelling. Displacement ellipsoids are drawn at the 50% probability level.

**Fig. 2.**
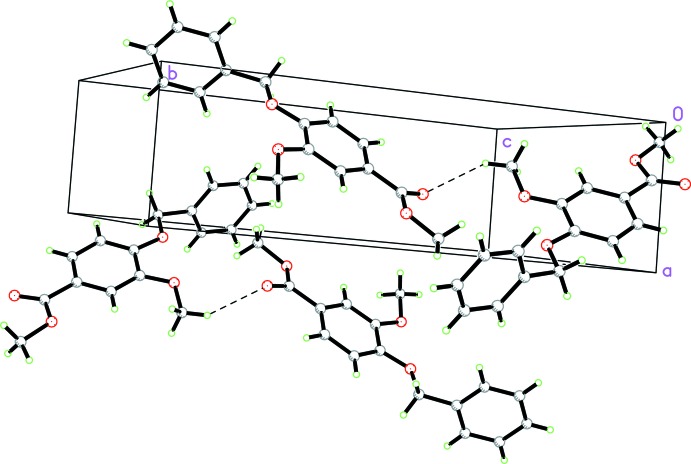
A view of the crystal packing of the title compound. The C—H···O hydrogen bonds are shown by dashed lines (see Table 1 for details; H atoms not involved in hydrogen bonding have been omitted for clarity).

## supplementary crystallographic information

### 1. Comment

The title compound is an important organic intermediate which has been used to synthesis the antineoplastic drug Cediranib. The drug has shown promising activity against diseases which include lung and breast cancer (Folkman, 1996).

The molecular structure of the title molecule is shown in Fig. 1. The bond lengths (Allen *et al.*, 1987) and angles are within normal ranges. In the molecule the two aromatic rings (C1-C6) and (C8-C13) are almost normal to one another with a dihedral angle of 85.81 (10) °.

In the crystal, molecules are linked by C—H···O hydrogen bonds forming chains propagating along the b axis direction (Table 1 and Fig. 2). There are also C-H···π interactions present (Table 1) linking the chains to form two-dimensional networks lying parallel to (102).

### 2. Experimental

The title compound was prepared according to the procedure reported by Li & Zhang (2012). A solution of 4-(benzyloxy)-3-methoxybenzoic acid (5 g, 19.36 mmol) was added slowly to a solution of methanol and concentrated sulfuric acid (2 ml). After being stirred for 12 h at reflux, saturated sodium bicarbonate solution was added to adjust the pH to 7. Dichloromethane was added, and the mixture was then filtered and the organic phase evaporated on a rotary evaporator and to obtain the title compound. Block-like colourless crystals were obtained by dissolving (0.5 g, 1.84 mmol) of the title compound in ethanol (25 ml) and evaporating the solvent slowly at room temperature for about 7 days.

### 3. Refinement

All the H atoms were positioned geometrically and constrained to ride on their parent atom: C—H = 0.93 - 0.96 Å with *U*_iso_(H) = 1.5*U*_eq_(C-methyl) and = 1.2*U*_eq_(C) for other H atoms.

### Crystal data

**Table:**

C_16_H_16_O_4_	*F*(000) = 576
*M**_r_* = 272.29	*D*_x_ = 1.292 Mg m^−^^3^
Monoclinic, *P*2_1_/*c*	Mo *K*α radiation, λ = 0.71073 Å
Hall symbol: -P 2ybc	Cell parameters from 1683 reflections
*a* = 5.2466 (7) Å	θ = 5.3–45.5°
*b* = 17.973 (2) Å	µ = 0.09 mm^−^^1^
*c* = 14.8785 (18) Å	*T* = 293 K
β = 94.018 (3)°	Block, colourless
*V* = 1399.6 (3) Å^3^	0.12 × 0.12 × 0.10 mm
*Z* = 4	

### Data collection

**Table:**

Enraf–Nonius CAD-4 diffractometer	1666 reflections with *I* > 2σ(*I*)
Radiation source: fine-focus sealed tube	*R*_int_ = 0.029
Graphite monochromator	θ_max_ = 25.0°, θ_min_ = 2.3°
ω/2θ scans	*h* = −6→6
Absorption correction: ψ scan (North *et al.*, 1968)	*k* = −21→14
*T*_min_ = 0.989, *T*_max_ = 0.991	*l* = −16→17
7732 measured reflections	3 standard reflections every 120 min
2454 independent reflections	intensity decay: 1%

### Refinement

**Table:**

Refinement on *F*^2^	Primary atom site location: structure-invariant direct methods
Least-squares matrix: full	Secondary atom site location: difference Fourier map
*R*[*F*^2^ > 2σ(*F*^2^)] = 0.045	Hydrogen site location: inferred from neighbouring sites
*wR*(*F*^2^) = 0.138	H-atom parameters constrained
*S* = 0.91	*w* = 1/[σ^2^(*F*_o_^2^) + (0.0874*P*)^2^ + 0.101*P*] where *P* = (*F*_o_^2^ + 2*F*_c_^2^)/3
2454 reflections	(Δ/σ)_max_ = 0.005
182 parameters	Δρ_max_ = 0.18 e Å^−^^3^
0 restraints	Δρ_min_ = −0.17 e Å^−^^3^

### Special details

**Table:**

Geometry. All e.s.d.'s (except the e.s.d. in the dihedral angle between two l.s. planes) are estimated using the full covariance matrix. The cell e.s.d.'s are taken into account individually in the estimation of e.s.d.'s in distances, angles and torsion angles; correlations between e.s.d.'s in cell parameters are only used when they are defined by crystal symmetry. An approximate (isotropic) treatment of cell e.s.d.'s is used for estimating e.s.d.'s involving l.s. planes.
Refinement. Refinement of *F*^2^ against ALL reflections. The weighted *R*-factor *wR* and goodness of fit *S* are based on *F*^2^, conventional *R*-factors *R* are based on *F*, with *F* set to zero for negative *F*^2^. The threshold expression of *F*^2^ > σ(*F*^2^) is used only for calculating *R*-factors(gt) *etc*. and is not relevant to the choice of reflections for refinement. *R*-factors based on *F*^2^ are statistically about twice as large as those based on *F*, and *R*- factors based on ALL data will be even larger.

### Fractional atomic coordinates and isotropic or equivalent isotropic displacement parameters (Å^2^)

**Table:**

	*x*	*y*	*z*	*U*_iso_*/*U*_eq_	
O1	0.8880 (3)	0.30300 (7)	0.59482 (9)	0.0538 (4)	
O2	0.5720 (3)	0.29367 (7)	0.71794 (9)	0.0573 (4)	
O3	0.3782 (4)	0.61921 (8)	0.64985 (12)	0.0841 (5)	
O4	0.2344 (3)	0.55257 (8)	0.76146 (10)	0.0702 (5)	
C1	1.3108 (4)	0.18726 (11)	0.53747 (14)	0.0585 (6)	
H1	1.4248	0.2124	0.5774	0.070*	
C2	1.3529 (5)	0.11390 (13)	0.51813 (17)	0.0714 (7)	
H2	1.4940	0.0896	0.5457	0.086*	
C3	1.1895 (5)	0.07597 (12)	0.45856 (17)	0.0703 (7)	
H3	1.2194	0.0263	0.4453	0.084*	
C4	0.9838 (5)	0.11185 (14)	0.41926 (18)	0.0789 (7)	
H4	0.8709	0.0866	0.3790	0.095*	
C5	0.9409 (4)	0.18540 (14)	0.43850 (16)	0.0682 (6)	
H5	0.8002	0.2095	0.4104	0.082*	
C6	1.1025 (4)	0.22385 (11)	0.49856 (13)	0.0475 (5)	
C7	1.0484 (4)	0.30341 (11)	0.52037 (14)	0.0552 (5)	
H7A	1.2064	0.3297	0.5366	0.066*	
H7B	0.9622	0.3279	0.4687	0.066*	
C8	0.7756 (3)	0.36807 (10)	0.61691 (12)	0.0456 (5)	
C9	0.6001 (3)	0.36283 (10)	0.68347 (12)	0.0430 (4)	
C10	0.4714 (3)	0.42556 (10)	0.70828 (12)	0.0463 (5)	
H10	0.3552	0.4222	0.7525	0.056*	
C11	0.5133 (4)	0.49364 (10)	0.66796 (12)	0.0481 (5)	
C12	0.6864 (4)	0.49822 (11)	0.60332 (15)	0.0629 (6)	
H12	0.7146	0.5438	0.5760	0.075*	
C13	0.8196 (4)	0.43625 (11)	0.57813 (16)	0.0631 (6)	
H13	0.9389	0.4404	0.5350	0.076*	
C14	0.3712 (4)	0.28318 (11)	0.77630 (14)	0.0581 (6)	
H14A	0.3996	0.3146	0.8282	0.087*	
H14B	0.3679	0.2321	0.7951	0.087*	
H14C	0.2109	0.2958	0.7450	0.087*	
C15	0.3720 (4)	0.56143 (11)	0.69069 (15)	0.0561 (5)	
C16	0.0836 (5)	0.61549 (13)	0.78568 (19)	0.0836 (8)	
H16A	0.1948	0.6546	0.8085	0.100*	
H16B	−0.0272	0.6009	0.8312	0.100*	
H16C	−0.0171	0.6329	0.7335	0.100*	

### Atomic displacement parameters (Å^2^)

**Table:**

	*U*^11^	*U*^22^	*U*^33^	*U*^12^	*U*^13^	*U*^23^
O1	0.0645 (9)	0.0414 (7)	0.0585 (8)	0.0127 (6)	0.0255 (7)	0.0029 (6)
O2	0.0783 (10)	0.0372 (7)	0.0597 (8)	0.0080 (6)	0.0284 (7)	0.0058 (6)
O3	0.1240 (15)	0.0404 (9)	0.0900 (12)	0.0199 (8)	0.0231 (10)	0.0051 (8)
O4	0.0835 (11)	0.0497 (9)	0.0804 (10)	0.0211 (7)	0.0277 (9)	−0.0038 (7)
C1	0.0590 (13)	0.0555 (13)	0.0617 (13)	0.0077 (10)	0.0084 (10)	−0.0034 (10)
C2	0.0729 (15)	0.0581 (14)	0.0848 (17)	0.0183 (12)	0.0168 (13)	0.0063 (13)
C3	0.0827 (17)	0.0462 (12)	0.0868 (17)	−0.0010 (12)	0.0399 (14)	−0.0076 (12)
C4	0.0712 (16)	0.0758 (18)	0.0911 (18)	−0.0194 (13)	0.0155 (14)	−0.0256 (14)
C5	0.0511 (12)	0.0743 (16)	0.0796 (16)	0.0061 (11)	0.0058 (11)	−0.0059 (13)
C6	0.0469 (11)	0.0491 (11)	0.0487 (11)	0.0023 (9)	0.0188 (9)	0.0002 (9)
C7	0.0582 (12)	0.0511 (12)	0.0591 (12)	0.0094 (9)	0.0242 (10)	0.0056 (10)
C8	0.0485 (10)	0.0385 (10)	0.0504 (10)	0.0063 (8)	0.0082 (8)	−0.0017 (8)
C9	0.0503 (10)	0.0344 (9)	0.0448 (10)	0.0026 (8)	0.0062 (8)	0.0007 (8)
C10	0.0510 (11)	0.0433 (11)	0.0456 (10)	0.0046 (8)	0.0097 (8)	−0.0038 (9)
C11	0.0548 (11)	0.0358 (10)	0.0538 (11)	0.0039 (8)	0.0054 (9)	−0.0034 (9)
C12	0.0768 (14)	0.0374 (10)	0.0771 (15)	0.0008 (10)	0.0244 (12)	0.0060 (10)
C13	0.0676 (13)	0.0468 (12)	0.0786 (14)	0.0061 (10)	0.0323 (11)	0.0080 (11)
C14	0.0702 (14)	0.0482 (12)	0.0581 (12)	−0.0017 (10)	0.0208 (10)	0.0073 (10)
C15	0.0660 (13)	0.0408 (11)	0.0613 (13)	0.0066 (9)	0.0027 (11)	−0.0086 (10)
C16	0.0882 (18)	0.0625 (15)	0.1027 (19)	0.0262 (13)	0.0258 (15)	−0.0171 (14)

### Geometric parameters (Å, º)

**Table:**

O1—C8	1.360 (2)	C7—H7A	0.9700
O1—C7	1.437 (2)	C7—H7B	0.9700
O2—C9	1.357 (2)	C8—C13	1.381 (3)
O2—C14	1.424 (2)	C8—C9	1.402 (3)
O3—C15	1.205 (2)	C9—C10	1.378 (2)
O4—C15	1.327 (3)	C10—C11	1.387 (3)
O4—C16	1.440 (2)	C10—H10	0.9300
C1—C6	1.369 (3)	C11—C12	1.371 (3)
C1—C2	1.371 (3)	C11—C15	1.477 (3)
C1—H1	0.9300	C12—C13	1.381 (3)
C2—C3	1.371 (3)	C12—H12	0.9300
C2—H2	0.9300	C13—H13	0.9300
C3—C4	1.354 (3)	C14—H14A	0.9600
C3—H3	0.9300	C14—H14B	0.9600
C4—C5	1.374 (3)	C14—H14C	0.9600
C4—H4	0.9300	C16—H16A	0.9601
C5—C6	1.374 (3)	C16—H16B	0.9601
C5—H5	0.9300	C16—H16C	0.9600
C6—C7	1.498 (3)		
			
C8—O1—C7	117.96 (14)	O2—C9—C10	125.47 (16)
C9—O2—C14	117.16 (14)	O2—C9—C8	115.00 (15)
C15—O4—C16	116.30 (17)	C10—C9—C8	119.53 (16)
C6—C1—C2	120.6 (2)	C9—C10—C11	120.70 (17)
C6—C1—H1	119.7	C9—C10—H10	119.7
C2—C1—H1	119.7	C11—C10—H10	119.7
C1—C2—C3	120.8 (2)	C12—C11—C10	119.29 (17)
C1—C2—H2	119.6	C12—C11—C15	118.61 (17)
C3—C2—H2	119.6	C10—C11—C15	122.08 (18)
C4—C3—C2	119.1 (2)	C11—C12—C13	120.99 (18)
C4—C3—H3	120.5	C11—C12—H12	119.5
C2—C3—H3	120.5	C13—C12—H12	119.5
C3—C4—C5	120.3 (2)	C12—C13—C8	120.00 (19)
C3—C4—H4	119.8	C12—C13—H13	120.0
C5—C4—H4	119.8	C8—C13—H13	120.0
C4—C5—C6	121.1 (2)	O2—C14—H14A	109.5
C4—C5—H5	119.4	O2—C14—H14B	109.5
C6—C5—H5	119.4	H14A—C14—H14B	109.5
C1—C6—C5	118.12 (19)	O2—C14—H14C	109.5
C1—C6—C7	121.67 (18)	H14A—C14—H14C	109.5
C5—C6—C7	120.21 (18)	H14B—C14—H14C	109.5
O1—C7—C6	106.99 (15)	O3—C15—O4	122.60 (18)
O1—C7—H7A	110.3	O3—C15—C11	124.3 (2)
C6—C7—H7A	110.3	O4—C15—C11	113.09 (18)
O1—C7—H7B	110.3	O4—C16—H16A	109.5
C6—C7—H7B	110.3	O4—C16—H16B	109.4
H7A—C7—H7B	108.6	H16A—C16—H16B	109.5
O1—C8—C13	125.04 (17)	O4—C16—H16C	109.5
O1—C8—C9	115.48 (15)	H16A—C16—H16C	109.5
C13—C8—C9	119.47 (16)	H16B—C16—H16C	109.5
			
C6—C1—C2—C3	0.9 (3)	O1—C8—C9—C10	−178.12 (16)
C1—C2—C3—C4	−0.5 (3)	C13—C8—C9—C10	0.9 (3)
C2—C3—C4—C5	0.4 (4)	O2—C9—C10—C11	−179.44 (17)
C3—C4—C5—C6	−0.9 (4)	C8—C9—C10—C11	0.2 (3)
C2—C1—C6—C5	−1.3 (3)	C9—C10—C11—C12	−0.5 (3)
C2—C1—C6—C7	178.4 (2)	C9—C10—C11—C15	177.82 (18)
C4—C5—C6—C1	1.3 (3)	C10—C11—C12—C13	−0.2 (3)
C4—C5—C6—C7	−178.4 (2)	C15—C11—C12—C13	−178.6 (2)
C8—O1—C7—C6	−168.61 (16)	C11—C12—C13—C8	1.3 (4)
C1—C6—C7—O1	−90.8 (2)	O1—C8—C13—C12	177.3 (2)
C5—C6—C7—O1	88.9 (2)	C9—C8—C13—C12	−1.6 (3)
C7—O1—C8—C13	−5.6 (3)	C16—O4—C15—O3	2.0 (3)
C7—O1—C8—C9	173.40 (16)	C16—O4—C15—C11	−177.77 (18)
C14—O2—C9—C10	8.2 (3)	C12—C11—C15—O3	8.1 (3)
C14—O2—C9—C8	−171.46 (16)	C10—C11—C15—O3	−170.3 (2)
O1—C8—C9—O2	1.5 (2)	C12—C11—C15—O4	−172.12 (18)
C13—C8—C9—O2	−179.44 (18)	C10—C11—C15—O4	9.5 (3)

### Hydrogen-bond geometry (Å, º)

Cg is the centroid of the C1–C6 ring

**Table:**

*D*—H···*A*	*D*—H	H···*A*	*D*···*A*	*D*—H···*A*
C14—H14*B*···O3^i^	0.96	2.53	3.379 (3)	147
C14—H14*A*···*Cg*^ii^	0.96	2.75	3.519 (2)	137

Symmetry codes: (i) −*x*+1, *y*−1/2, −*z*+3/2; (ii) *x*−1, −*y*−1/2, *z*−1/2.
